# A tale of be(com)ing a Sport Nutritionist: Reflexive insights from a researcher-practitioner entering the field

**DOI:** 10.1371/journal.pone.0319164

**Published:** 2025-02-27

**Authors:** Meghan R.N. Bentley, Laurie B. Patterson, Susan H. Backhouse

**Affiliations:** Carnegie School of Sport, Leeds Beckett University, Leeds, United Kingdom; King Faisal University, SAUDI ARABIA

## Abstract

Qualitative research can facilitate an understanding of the richness and complexity of human experience, shedding light on multifaceted relationships that exist within sporting environments. Within the field of sport nutrition, such immersive methods are not commonplace. Yet could offer a significant contribution to our understanding and help practitioners navigate their values and identity within the challenging world of high-performance sport. The first author, a neophyte sport nutrition researcher-practitioner, reflects upon her experience over a four-year period of integrating into a high-performance organisation and embarking upon a research programme and career as a sport nutritionist. Drawing on field notes, reflexive journal entries, and regular research team discussions, three moments of discovery are shared: 1) Navigating the transition into high-performance sport, 2) Understanding the performance pressures and emotional burden experienced by athletes, and 3) Negotiating collaboration in the nutritionist-athlete relationship. Through this confessional tale, we recognise the development of a sport nutritionist’s values and identity is unlikely to be smooth or linear. Instead, it may be a bumpy voyage of self-inquiry and discovery, shaped by diverse cultural experiences. To help future sport nutrition researcher-practitioners entering the field, we offer three meaningful development activities including 1) engaging in reflexive practice with critical friends to explore the dilemmas and uncertainties that may impact personal and professional development, 2) fostering intellectual candour when engaging in reflexive practice to facilitate self-discovery and growth, and 3) prioritise reading the growing literature on sport and organisational culture to inform and influence the delivery of sport nutrition practice.

## Introduction

Over the past 50 years or so the research focus of sport nutrition has been situated within the positivist paradigm, whereby knowledge is viewed as observable, empirical, quantifiable, and verifiable [1]. As such, quantitative research has served to establish and confirm nutritional strategies (e.g., pre-exercise fuelling recommendations) and physiological theories (e.g., that exogenous carbohydrate ingestion delays fatigue and mammalian target of rapamycin (mTOR) pathway regulates protein synthesis) [2–4]. Whilst this paradigm has helped to generate the evidence-base that underpins *what* nutritional guidelines are provided [5], it assumes athletes are a “closed system”, causality is linear, and that subjective experience is inconsequential [6]. Therefore, whilst the focus of the field has been on establishing the “what”, we are yet to develop our understanding of *how* nutritional recommendations are delivered within high-performance sport.

In recent years, calls have been made across academic fields to embrace an interpretivist paradigm [e.g., 7] in which knowledge is viewed as multiple, situated, and socially and historically bounded. In response, research questions which acknowledge human experiences, such as, “how do athletes experience nutritional adherence in high-performance sport?” would be investigated. This type of research question, underpinned by qualitative methodology, allows us to understand the world from the participant’s point of view and facilitates a more in-depth understanding of the richness, complexity, and multifaceted relationships within sporting environments [for examples of such work, see 8–10]. Therefore, acknowledging athletes’ experiences has the potential to offer new understanding which can shape sport nutrition services and/or interventions that are targeted and tailored to athletes’ needs, and in turn, optimised to protect athlete health and wellbeing, and enhance performance [11,12].

Despite the potential for athlete insights to improve sport nutrition services, it is not routine practice to include athletes in the design and development of a sport nutrition service. A systematic review of sport nutrition interventions noted that the design and development of all 16 eligible interventions was not informed by a systematic behavioural analysis [13]. A behavioural analysis is when researchers and/or practitioners take time to understand athletes’ experiences and behaviours from their point of view and identify the barriers and enablers that need to be targeted through a behavioural intervention [14]. Instead, sport nutrition behavioural interventions are typically based on the assumptions and *perceived* barriers to change of the intervention developers/practitioners. In such instances, we might argue that sport nutrition interventions are conducted *on* athletes rather than designed and delivered in partnership *with* athletes [15]. The value of working in partnership *with* those the intervention is serving has been advocated by multiple disciplines (e.g., sports psychology, public health, and physical activity). Adopting this approach allows those being impacted by an intervention to usefully shape, contribute to, and benefit from research [15]. Specifically, nesting intervention development within co-production helps to ensure a nutritional service and/or intervention considers the direct needs of the athlete group that it intends to serve.

In addition to a lack of athlete-centred approaches to sport nutrition delivery, very few sport nutrition interventions account for the range of factors that influence human behaviour. Specifically, a systematic review noted that only 3 of the 16 eligible sport nutrition interventions they reviewed used a behavioural theory to comprehensively guide intervention design, development, and implementation [13]. Yet, a theoretical application is strongly advocated within the UK’s Medical Research Council guidance on developing and implementing behavioural interventions [16]. Given the recommendations to draw on behavioural theory during intervention design and development, the Capability, Opportunity, and Motivation Behaviour (COM- B) model [17] was deemed helpful to underpin the first author’s programme of doctoral study. In comparison to pre-existing behaviour change theories, the COM-B model acknowledges the significance of automatic processes (e.g., emotions and impulses) alongside reflective cognitive processes (e.g., beliefs and intentions), and shifts our focus from individual cognition (e.g., it is the athletes’ responsibility to change their thoughts and beliefs) to a broader perspective which underlines the importance of the physical and social environment in shaping human behaviour (i.e., it is also the responsibility of the environment to change its physical and social form to better support athletes’ behaviour). The COM-B model, therefore, helps to illuminate the complexity of human behaviour, which is affected by a myriad of intrapersonal, interpersonal, and external factors.

It is well acknowledged within cultural sport psychology that human experience and behaviour are embedded in the culture and social milieu we inhabit [18]. Culture is complex and multifaceted, and it is influenced by various factors such as history, leadership, and the external environment [19]. Typically, culture is thought to encompass the behaviours, values, expectations, attitudes, and norms of a collective. Thinking about organisational culture specifically, Schein [19] asserted that it consists of three layers: 1) underlying assumptions and beliefs (that may be conscious or unconscious), 2) norms and values about appropriate attitudes and behaviours (that may be espoused or real), and 3) cultural artefacts that may reflect these (e.g., clothing, buildings, stories told in the environment, symbols displayed).

There is growing recognition that in order to fully understand human experience and behaviour, there must be an understanding of the cultures and environments in which an individual exists [20,21]. High-performance sport is described as an inherently competitive environment [22] which operates under distinct performance policies, selection procedures, and rigid training and competition schedules. It has been posited that “high-performing cultures prevail when the shared perception and action of elite team environment members (a) supports sustained optimal performance; (b) persists across time in the face of variable results (i.e., wins, losses, ties); and, most importantly; (c) leads to consistent high-performance” [23, p.340]. To date, our understanding of how these interactive effects within high-performance sport settings shape athlete adherence to nutritional guidance/recommendations is limited. Given the high prevalence of Low Energy Availability (LEA)/Relative Energy Deficiency in sport (REDs) among athletes [24], and the importance of dietary behaviour in preventing LEA/REDs, understanding athletes’ dietary behaviour is a relevant, timely, and significant behavioural problem to explore.

To understand the interactions between culture, human experience, and behaviour, recent work has recommended a methodological practice in which the researcher changes from an objective observer to a participant [25]. Researchers utilising such participatory practices may conduct immersive field work to facilitate a more in-depth understanding of the complexities within natural environments [26]. Immersive methods are useful in enabling researchers to acquire an understanding of issues where research is either sparse or non-existent [27] and it allows the researcher to learn what is it like to be situated in a new environment or community [28]. Immersive methods evoke the ‘joining of two worlds’, in which the perspectives of both the researcher and the researched are combined, exposing news ways of seeing things and exposing new things to see [29]. In high-performance sport settings athletes maintain consistent contact with the environment, through their training commitments and competition endeavours, and staff have an intimate involvement within athletes’ lives. Therefore the researcher, and the researched population, are all intertwined within the research process [28]. Consequently, the researcher is an “active” instrument who is a part of the research findings and not separated from them [30]. Accordingly, researchers must take into serious consideration their mindful body, that obviously and inevitably is present in the research process [31].

To our knowledge, there have been no published studies within sport nutrition that have made use of immersive qualitative methods within high-performance environments. Additionally, there is a lack of guidance for sport nutrition research-practitioners seeking to conduct qualitative research within the sporting context. Such guidelines and methods are beneficial and offer value for investigations seeking to advance sport nutrition professional practice and a holistic understanding of “high-performance” sport cultures. As a doctoral student and neophyte sport nutrition practitioner embarking upon a doctoral research programme and career as a sport nutritionist, I (the first author (MB)) sometimes felt lost and confused due to a lack of research shining a light on the experience of research-practitioners conducting a behavioural analysis to whom I could relate to and learn from. Therefore, the aim of this paper is to foster reflexivity and provide a confessional tale of my experiences of using qualitative inquiry to understand athletes’ dietary behaviours within high-performance sport. Specifically, this paper seeks to address the following objectives:

1) To document the experience of reflexive practice in action by a sport nutrition researcher-practitioner;
2) To provide an insight into the challenges of becoming an immersed sport nutrition researcher-practitioner conducting qualitative inquiry;
3) To explore how organisational culture and experiences shape practitioner values and identity.

To accomplish our aims and objectives, we first present the research context and our positioning as researcher-practitioners. We then highlight the relevance of reflective and reflexive practice and provide information on data collection, data analysis, and research quality.

## Methodology

### Confessional tale

This paper uses the genre of a confessional tale to explore the research process as experienced by the first author who occupied a dual role as a sport nutrition researcher-practitioner. This confessional tale stands alongside and extends our recently published realist tales which explored athletes’ and sport nutritionists’ experiences of nutritional adherence within high-performance sport [32,33]. Researcher-practitioner experiences are sparse within sport nutrition, meaning the reality of fieldwork is often left undocumented [30]. However, confessional tales are increasingly being used in sport psychology to understand the research process as experienced by the author(s) [For examples of such work, see 21,26,28,34,35]. Adopting an immensely personalised style, confessional tales are a method for researchers to discuss the dilemmas and revelations they experienced whilst embedded in applied settings [30,36]. Consequently, we view this article as the opportunity for the first author (I) to position herself at the forefront of this piece, announcing; this is what I experienced, and this is how I felt. As a research team, we hope that this article will illuminate the deeply complex and interactive nature of role, values, and identity of the first author as an embedded sport nutrition researcher-practitioner. Furthermore, our ambition as a research team is to generate knowledge that can make a positive difference to people, communities, and organisations. Thus, we aspire to inform the education and supervision of students and early career practitioners to help them navigate career challenges and their practitioner values and identity within the challenging world of high-performance sport.

### Philosophical positioning

This confessional tale was guided by our relativist ontology and subjectivist epistemology. This positioning states social reality is humanly constructed and shaped in ways that make it fluid and multifaceted [25]. Therefore as researchers we acknowledge that multiple subjective realities exist [25]. In relation to epistemology, we believe researchers are an inseparable part of what is being studied, meaning “reality” does not exist independent of us [37].

### Reflective and reflexive practice

Reflexivity is the common characteristic underpinning confessional tales, as it is the main instrument for gathering, interpreting, and presenting “data” as stories of lived experience [38]. However, the term “reflection” and “reflexivity” are often used interchangeably despite critical differences. Reflection can be considered a process of in-depth consideration of events outside of oneself [39]. For example, drawing on influential work within the education literature, knowledge or “professional artistry” can be developed by engaging in two processes of *reflection-in-action* and *reflection-on-action* [40]. Reflexivity moves beyond reflection as a process and involves a critical exploration of how our own life experiences and contexts (which are fluid and changing) impacts and informs the process and outcome of knowledge generation [41]. Researchers consider reflexivity to consist of two main types, prospective and retrospective [42]. Prospective reflexivity seeks to help researcher-practitioners understand how the knowledge, feelings, and values that they bring into the field impacts the findings [43]. In comparison, retrospective reflexivity concerns the effect that the research has on the researcher, and how the intertwined experiences can shape the interpretation of phenomena [43]. In this research, I seek to be reflective and both prospectively and retrospectively reflexive.

### Research context and biographical positioning

My doctoral research programme was established in partnership with Leeds Beckett University and a sports organisation. The supervisory team and leaders of the sports organisation had a shared interest in human behaviour and working in collaborative ways with sport nutritionists and athletes to develop professional practice. Within this paper, we represent the situated sports organisation using the title High-Performance Organisation (HPO). HPO is a grant-funded organisation providing sport science and medicine support services to national governing bodies. Within the HPO athletes received funding to participate in their sport, the amount of funding an athlete received was determined by the level of performance the athlete has achieved and the level they are thought to be capable of producing in the future.

When I began the research programme, my experience as a sport nutritionist and my knowledge and awareness of the high-performance sporting context was limited. My educational background was in public health nutrition which focused on the promotion of food for health and prioritised client autonomy through person-centred counselling approaches. My first encounter with high-performance sport came during the first year of my doctoral research programme, where I joined a large team of performance nutritionists working across multiple sports and I worked as a junior nutritionist for a Paralympic sport (referred to as *Para Sport* throughout this paper). This was my first position as a practitioner, where I was contracted to one day per week and held sole responsibility for the delivery of nutritional support to athletes. Methods of support included nutritional screening, individual support sessions, group workshops, and staff education. Throughout my doctoral research programme co-authors LP and SB provided academic supervision for my programme of research and I had a professional practice supervisor within HPO.

### Data collection

This piece is framed by the experiences I had as an embedded sport nutrition researcher-practitioner within HPO across a four-year period. The longitudinal nature of the study allowed me to capture my journey to be(com)ing a sport nutrition researcher-practitioner. At the initial stages of my researcher-practitioner endeavour, I primarily relied on *reflection-in-action* [40], which entailed *in-vivo* fieldwork reflections. Specifically, on every occasion I was present within the organisation I made field notes which included brief sentences or phrases to identify key events and their details (e.g., what happened, where, and what was said, by whom). This included observations of training and competitions, meetings (with staff and athletes), training camps, and formal and informal staff outings. As my familiarity with the organisation and the culture grew, so did my reflections. Subsequently, my field notes evolved into a reflexive journal whereby I captured my thoughts, feelings, and ideas, which helped me make sense of my experiences and consider the impact of such events on my practitioner values and identity. To elaborate, within my reflective journal of over 100 pages and 25,000 words, I questioned my own assumptions, examined my worldview juxtaposed with the athletes and staff with whom I interacted, and expressed the ideas and ruminations that I had. Moreover, I reflected on meaningful interactions with athletes and staff through reflexive discussions with my supervisory team. The supervisory team did not have exposure to the research setting and this physical distance fostered a rich collaborative dialogue which helped me to engage in more in-depth reflections when making sense of my experiences throughout the research process. Taking the time to write in-depth reflections further engrained them into my memory and served as the primary tool for the creation of meaning prior to more formal analysis.

### Data analysis

The first step of data analysis was to review my field notes and reflexive journal which captured my journey across time. Consequently, the events that had a significant impact on my journey to be(com)ing a sport nutrition researcher-practitioner were presented as a timeline. From this timeline, I adopted the stance of a *story analyst*, in which I engaged in rigorous analysis of the narrative [44]. To elaborate, the data capturing significant events were analysed through a social constructionist lens using a reflexive thematic analysis (TA) approach [45]. Specifically, I engaged in in-dwelling to increase familiarity with the content by reading and re-reading the timeline of significant events (Stage one). During this process I recorded my initial impressions to capture meaningful patterns. Subsequently, I carried out coding using pen and paper (Stage two), which were then clustered into candidate themes and supported by compelling quotes form my reflexive journal (Stage three). At this stage, I engaged in collaborative and reflexive TA with my supervisory team whereby themes were critically discussed in order to develop a richer and more nuanced understanding of the data (Stages four and five) [46]. We recognise that our interpretations are contextually bounded, positioned, and situated [47], therefore the aim was to understand and create meaning from my experiences as opposed to identifying an objective and singular truth. Recognising that writing is a form of analysis [30] and a part of the analytical process (Stage six) [45], I endorse the standpoint of a *story sharer*, who aspires to *show* (rather than *tell)* theory *in* and *through* a story by using confessional tale as a creative analytical practice [48].

### Research quality

Aligned with the relativist approach [25], the reader is encouraged to judge this research using the following criteria: (a) rich rigor, (b) credibility, (c) resonance, (d) significant contribution, and (e) ethics. As a measure of *rich rigor*, prolonged immersion (four years) within the research setting facilitated the building of rapport and development of trusting relationships with participants (athletes and staff). Rigor was also assured by providing in-depth and transparent descriptions of the methods used during data collection and analysis. Moreover, I engaged in dialogue with critical friends (i.e., my supervisory team) who repeatedly prompted reflexivity and regularly challenged the interpretations of the data by encouraging alternative explanations for the data gathered. To achieve *credibility*, thick description of the research context has been provided to enable readers to account for the specificity and circumstantiality of the data. For example, the presentation of real-life descriptions and the quotes from my reflexive journal aim to provide abundant and robust detail. Regarding *resonance,* I have provided detailed description of my life and the lives of participants through “evidence”, contextual details, and richly layered theoretical expressions, which seeks to enhance the likelihood that the findings meaningfully reverberate and affect the audience [49]. Given its novelty, this study makes a *significant contribution* to knowledge, ideas, and methodological approaches within sport nutrition, which we hope has a lasting influence on the field. Finally, in terms of *ethics,* ethical approval for the programme of research was obtained from Leeds Beckett University ethics committee (Ref: 35405, 48996). All participants gave written informed consent using a consent form which was witnessed by the lead researcher (MB) and the recruitment for the focus groups and interviews was between 28/04/2017 and 24/08/2018. To assure confidentiality, the name and location of the organisation is not disclosed and no real names or information that might lead to the identification of any individual included. Due to the nature of qualitative research being an ongoing interaction, informed consent was considered an ongoing process of construction and negotiation [25]. For example, throughout the study I updated participants on how the research was unfolding and re-established consent. However, given the specificity of the research context and occupying a dual role as a researcher-practitioner within the organisation, I faced conflict between conveying rich description and protecting the identity of the organisation and individuals within it. As a result, I found it helpful to draw upon key tenants of relational ethics [50]. Accordingly, throughout this research journey I based my actions and behaviours on my relations and commitment to athletes and staff within HPO. Specifically, I aimed to ensure that I engaged in open dialogue and active listening with participants and connected with others based on valuing participants dignity and having (and demonstrating) mutual respect [51].

## Results and discussion

In this section, we present reflections on three moments of discovery including 1) Navigating the transition into high-performance sport, 2) Understanding the performance pressures and emotional burden experienced by athletes, and 3) Negotiating collaboration in the nutritionist-athlete relationship. These aim to illuminate a) how the values that I brought into the research setting influenced my experiences and b) how the research process (namely engagement with the HPO cultural practices and the findings from our realist tales), challenged my ways of thinking about and working *with* athletes and staff; and ultimately my sport nutrition researcher-practitioner role, values, and identity.

### Navigating the transition into high-performance sport

On my first day in the HPO, which coincided with the start of a two-day continued professional development (CPD) event, I felt out of place and alone. During this event, it quickly became clear to me that members of this group had a wealth of experience and had established relationships with one another. Conversely, I was a young and inexperienced practitioner, naïve to the high-performance world and I did not know anybody, other than my manager, whom I had only recently met during the recruitment process for my role. I entered the organisation expecting to feel displaced and uncomfortable, however I had not comprehended how difficult it would be to be in an environment where everybody knew each other and were accustomed to the sport nutrition role and the high-performance sporting setting. To illustrate, the HPO included sport nutritionists who worked across multiple sporting environments with many practitioners having over 10 years of experience. I felt everybody knew something that I did not and were a part of something that I was not. I was an outsider. In one sense, I faced similar challenges to any employee entering an organisation for the first time. Indeed, many new recruits experience insecurity and self-doubt during the entry process [52–54]. For example, Sanders, Wadey [26] in their ethnographic study of an amputee rehabilitation clinic, acknowledged how unsettling it can be for a newcomer to join a hospital setting with no medical experience. Despite my feelings being *normal* and half-expected, the degree to which I experienced them was greater than I had imagined and had prepared myself for. My lack of sport nutrition practitioner experience meant I did not understand the unique emotional, social, and behavioural norms, practices, and conventions inherent to the high-performance sporting environments. Following my first interaction with the HPO nutrition team, I noted in my journal:

Being in this environment was exhausting and it was so awkward just stood there like a spare part. There were so many new people to meet, and I found I was constantly trying to start a conversation with someone or join in a conversation with others. But in these conversations, I had no experiences that I could draw on or relate to, and as result, I didn’t feel connected to anyone. I’ve left this event feeling like I have nothing worthy to contribute. Ultimately, I feel like I have met many people today, but I feel I do not know anyone, nor does anyone know me.

Researchers undergoing similar fieldwork have previously offered comparable findings of frustration, embarrassment, and burden when entering a new role as an outsider [e.g., 26,28] and have documented struggling to place themselves physically and emotionally alongside participants [e.g., 55]. Whilst I was not aware of this literature back then, it has brought comfort to me now by facilitating deeper reflexivity. Specifically, as I compare myself to others, I noticed I studied public health nutrition for my undergraduate degree and not sports science. As a result, I feared that my educational background did not prepare me for the high-performance setting or afford me an appreciation for performance compared to my peers. I therefore sought to learn more about the sport nutritionist role to “fit in” and be considered “good” at my job. Specifically, I discovered effective ways to engage with my fellow sport nutritionists was to help them within their roles. For example, I assisted a practitioner develop an athlete recipe book for a non-disability sport and I co-delivered workshops at training camps to non-para-athletes. By volunteering to support practitioners in their roles, I was able to observe their practice, and I began to build relationships. I wrote in my journal:

I feel as though I have made big strides with the HPO nutrition team today. Previously, whilst I said I wanted to learn about the role, there hadn’t been any obvious way in which I could do so. Whilst members of the team have always been polite and helpful, I don’t think they could see any way they could support me without me being a burden. Whereas now, I have identified multiple ways in which I can help them in their roles whilst also developing my understanding of the sport nutritionist’s role. I have since sensed a huge softening and acceptance towards me. Today arriving at a sport nutrition conference, my nutrition colleagues greeted me warmly with hugs and smiles, and I suddenly felt a part of the team.

Several months after joining the HPO, I began to feel less burdensome and more accepted as I moved from being an *observer as participant*, where I was an outsider to the HPO culture, to a *participant as observer,* where I was positioned on the inside of the HPO culture [25]. As a *participant as observer* I had access to the “backstage” behaviours normally inaccessible to “outsiders” [56]. For example, I observed sport nutrition consultations with non-disabled athletes and I had unrestricted access to high-performance training environments within non-disability sports. O’reilly [57, p. 112] argues, “participating enables the strange to become familiar and, observing enables the familiar to appear strange”. Specifically, during my observations and participation I learnt that 1) the role of a *sport nutritionist* was that of a *performance* nutritionist, 2) athletes’ training and competition performances were the focus of conversations, 3) what support could be delivered and when centred around the competition schedule, and 4) who received what support was decided by how “good” athletes’ performances were perceived to be. Influenced by the norms and cultural assets of the HPO, I adopted the title of a *performance* nutritionist when I started the sport nutrition role for Para Sport, with a singular focus on athletes’ performance. In other words, I advised athletes on what to eat to enhance their performance and I found means to reduce athlete burden so they can focus on their performance goals (i.e., by providing athletes with supplements and food).

Although I became more familiar with what I needed to do to gain experience within the role, and I felt privileged to be embedded in a high-performance sport and a team of sport nutritionists from whom I could learn, I remember feeling uncomfortable with my newfound identity as a *perfor*mance nutritionist. In particular, the expectation on and responsibility for athlete performance was at odds with my education and pre-existing values and identity. Specifically, my sense of self centred on the importance of food for health and the client’s need for autonomy towards their behavioural goals. The actions I was undertaking in this role were not what I had expected to be doing, and it felt like I was putting on a persona to fit in. For example, I was telling athletes what to do and how do it, rather than collaborating with athletes to understand what they want to do, and how they can do it for themselves. Yet, being a nutritionist in sport was all I had wanted to do. During this time of internal conflict, I remember doubting myself on countless occasions. For example, in my journal I wrote, “Maybe it’s me who does not understand the role”, “Maybe it’s me who needs to adjust to be better suited to the role and the environment in which it is situated”? After this period of rumination, I accepted the shared identity as a *performance* nutritionist, and I did not question or challenge the normative *way of being*. As such, I connected with the team through shared experiences and built trusting relationships along the way. As these relationships developed, I was able to explore my ruminations with them. This relational inquiry was particularly evident during the first qualitative interview study of my PhD, which occurred roughly six months after transitioning into the HPO, when rapport was developing. For example, I asked questions that explored commonly held assumptions (i.e., what is the role of a *performance* nutritionist? and what impact does a *performance* nutritionist have?). As a result, sport nutritionists shared in-depth and rich accounts of the challenges and dilemmas that they face within their roles [32].

Specifically, within Bentley et al., [32] sport nutritionists perceived that athletes are typically driven by external performance pressures (i.e., automatic motivation), rather than the intrinsic value of developing lifelong nutritional habits for health and wellbeing (automatic motivation). However, these performance pressures are reinforced by the beliefs and behaviours of the social influencers in the system (i.e., sports nutritionists and coaches) (social opportunity). The experiences shared was reassuring that I was not the only one who was uncertain on the role and influence as a sport nutritionist. In summary, it is important to acknowledge and illuminate the negotiations that sport nutrition researcher-practitioners might face when they first enter and operate within a high-performance environment. In the short-term adopting the cultural norms of the new environment might offset some of these challenges by enabling open dialogue with colleagues and other sport nutritionists. This in turn allows respectful exploration of any doubts that practitioners may have about their role through a mode of appreciative inquiry. Getting a sense of the history and cultural practices of a new organisation is certainly valuable for new practitioners.

### Understanding the performance pressures and emotional burden experienced by athletes

Ten months into my *performance* nutrition role within Para Sport, I undertook research with athletes to understand their experiences of nutritional adherence within high-performance sport. During these conversations, athletes provided rich insight into several emotional barriers they faced when following the nutritional guidance that is set by their *performance* nutritionist, including sadness, guilt, and shame. Intertwined with emotions and food, athletes illuminated the paradoxical role of performance in driving athletes’ motivation to adhere to nutritional guidance on the one hand, yet placing increased and often unbearable, demands on them on the other [33]. During data collection for the realist tale, I grew increasingly aware of how the athletes I was working with experienced performance pressures within the environment. Following a visit to Para Sport I noted in my journal:

Today at [training centre] a colleague expressed her concerns when highlighting the worries of one of the athletes on the programme, she shared; ‘they said to me yesterday [the athlete], “I know I’m running out of chances, and if I don’t show performance improvement over the next two comps, the sports funding is at risk, your job is at risk, everyone’s job is at risk”.’

Gaining first-hand experience of the performance pressures and emotional burden on athletes was pivotal in shaping my thoughts, feelings, and behaviours. My previous exposure to quantitative research in the field of sport nutrition (i.e., the physiological effects of particular nutrients) had done little to illuminate the emotional challenge and performance pressures that athletes experience. Throughout the fieldwork I remained aware of the possibility of a *food-for-performance* heuristic, and I endeavoured to explore my role in shaping and defining it. Specifically, I spent time reflecting on my behaviour in my reflexive journal and I engaged in reflective discussions with my supervisory team and multidisciplinary team of Para Sport. During this time my biases (e.g., athletes are motivated by performance, and this is a positive experience for all) and assumptions (e.g., it is the role of the sport nutritionist to do everything for athletes so they can focus on their performance) were challenged. I began to empathise with athletes on the demands of continuous performance targets and I became attentive to opportunities which facilitated athlete independence and autonomy with their nutrition.

This period of fieldwork provided valuable insight and feedback that enhanced my self-awareness as a researcher-practitioner. One afternoon during this period, a colleague in Para Sport (who will hereon be referred to as Harry) called me to discuss my role. During this conversation he asked, “how are you finding the role?” and “how are you getting on with the athletes?”. During this conversation, Harry shared that he was concerned with the quality of the relationships that I was developing with athletes. He felt I was rushing in to address their performance goals, not taking time to get to know them as people, nor focusing on their holistic development [58]. Whilst Harry’s ability to challenge me directly, yet show he also cares about me personally [59], was appreciated, the nature of the feedback was difficult to process. Subsequently I encountered a range of emotions that resulted in me questioning every aspect of my values and identity as a researcher-practitioner. It seemed I had been aligning to the culturally dominant and desirable performance narrative of the HPO. However, upon engaging in reflexive discussions with the supervisory team, I realised my entry into Para Sport was different to many of the staff and athletes in Para Sport. When I was finding my feet within the HPO, I interacted with a range of non-disability sporting environments which had each shaped my *performance* nutrition identity. Whereas, most of the staff and athletes in Para Sport had constructed their identity and meaning of sport within the Paralympic movement [60]—where human rights are core to decision making.

To elaborate, the philosophy that guides the Paralympic movement emphasises that *all athletes are human beings first* [61]. Consequently, participation, social inclusion through sport, and demonstrating what achievements are possible for disabled athletes is the focus rather than winning at all costs [61]. Therefore, athlete value and self-worth go far beyond their sporting performances and success is defined through holistic growth and development [61]. Following the conversation with Harry, I became acutely aware of this ethos and questioned whether Harry’s actions served as an indication that there is not just one culture in HPO but multiple micro-cultures operating within a wider system. A heightened sense of awareness ensued, and I became sensitive to the interactions I was having with athletes and staff, questioning how I conducted myself during those interactions. Harry’s candour was instrumental in developing my sport nutrition practice. For example, I started to take the time to understand the person behind the athlete by showing intrigue in their personal interests (beyond sport) and I put their wellbeing at the front and centre of our conversations. Furthermore, I encouraged athletes to actively engage in meetings and lead on their nutritional goals, and, with support from the wider team, I created opportunities that exposed athletes to developmental challenges (i.e., planning and preparing their own food and supplements for training and competitions).

Despite my interactions with Harry challenging my identity as a researcher-practitioner, over time and through engaging in reflective discussions with my supervisory team, the conversation with Harry was the catalyst that invited me to be the *sport* nutritionist whom I wanted to be. It encouraged me to ask questions about the persona I had created, as evidenced in my journal:

I have grown increasingly uncomfortable with the title: “Performance Nutritionist”. Reflecting on my experience and data, it feels inappropriate, limiting, and misleading to use a job title that only encompasses half of my role. What about athlete’s health and wellbeing? What about performance as a motivational driver having a maladaptive effect on athletes’ dietary behaviour? I feel the title of “Performance Nutritionist” may play a part in perpetuating the “performance narrative” by reflecting a ‘win at all costs’ culture that I do not identify with. Can I therefore choose to revert to the job title as I remember it, and as I had studied, “sport nutritionist”?

Managing these conflicting thoughts was challenging and I considered how I may have been complicit in the performance narrative, as I struggled to find my feet in a new and unfamiliar sporting environment. My imposter syndrome gave me a prevailing sense of uncertainty, which overshadowed my compassion for athletes and understanding their experiences of a stressful and competitive sporting environment. This notion is illuminated by McMahon and McGannon [62] study of athlete–medical practitioner relationships. They noted that doctors can act as abettors in performance ideologies, and as a result, compromise athlete health through the medical treatment they receive. It was at this point that I began to use every opportunity to signal to others that I identify as a *sport* nutritionist as opposed to a *performance* nutritionist. For example, I asked those in Para Sport to refer to me as a *sport* nutritionist, I changed my email signature, and I referred to myself as a *sport* nutritionist during presentations and when writing biographies for myself. In changing these cultural assets, I was signalling my professional identity, which is underpinned by my values and beliefs. These actions served to be important for my own personal and professional development.

### Negotiating collaboration in the nutritionist-athlete relationship

During the initial months at the HPO, I focused on personal integration into the setting and gave little consideration to interpersonal dynamics (and more specifically, how this might impact on the research process). As I became more familiar with the setting and my place within it, I was able to attend to the power dynamics between myself and athletes and staff. During data collection for the realist tales [32,33], I was an *outsider* coming into their sport for the first time. Consequently, I did not have a relationship with athletes, and I was worried a lack of trust would impact the quality of data obtained. I wrote in my journal:

I am conducting the first focus group with athletes tomorrow and I feel apprehensive. What will they think of me, a non-athlete, a *performance* nutritionist? How will they respond to the types of questions I ask? Will they be interested? Will they engage in the discussions? Ideally, I would have liked to have met them before they attend the focus group. However, this has not been possible.

As both a qualitative researcher and practitioner I recognise the potentially powerful position I occupied. Historically, researchers typically initiate and determine the topic of inquiry, and decide when to terminate conversation [63]. During the first focus group discussion, I found some athletes expected me to answer the questions posed and they often looked towards me for approval when they shared what they consumed to support their training and competitions. Literature on dyadic relationships in sport (e.g., coach-athlete) suggest unequal influence and power will always exist when one’s role is to “determine” what is good for the athlete concerning his or her sporting performance [64]. This role was being explicitly signalled in my job title, as I was introduced to athletes as a *performance* nutritionist and PhD *researcher* who worked at a university. Additionally, athletes were handed formal ethical documentation which was headed with the HPO and the university logo.

Throughout the field work I remained aware of the possibility of unequal power dynamics between myself as a researcher-practitioner and athletes. I endeavoured to guard against this disparity by creating a conversation style which allowed athletes to feel comfortable in disclosing honest feelings. For example, I aimed to reduce power imbalance by *setting the scene* at the beginning of the focus group by explaining to athletes that their input during the discussions was highly valued. I emphasised that there is an inherent interdependence between our role as sport nutritionists and their lived experiences; thus, speaking openly to one another is critical to inform the development of the nutrition service they are provided with. Secondly, I invited athletes to be open by explaining the importance of their knowledge and experiences and the value of their voice. I explained to athletes that they had agency and the power to change the nutritional service that is provided to them. But I reminded athletes that as sport nutritionists we have limited perspectives and through open communication, we need athletes to educate us on what life is like for them. Lastly, when athletes shared their thoughts, feelings, and experiences, I responded with gratitude. I did this by using phases such as, “that’s really insightful, thank you for sharing that” and “it’s so important for us to know and understand this, thank you for sharing”. By appreciating athletes’ willingness and courage to share their thoughts and opinions, I noticed athletes’ confidence grow throughout the discussions. As a result, athletes shared valuable knowledge and information about themselves. After facilitating the first focus group discussion, I noted in my journal:

Travelling back from the focus group I have this great sense of fulfilment. I’m surprised at how open and honest athletes are during these group discussions. I mean, this is the first time they have met me, yet they are so willing to share information that is personal and meaningful to them. At the end of the focus group I asked, “So…. how did you experience speaking about this topic with your teammates?”. An athlete turned to me, thanked me for the session and expressed gratitude for an opportunity to share their challenges with food and nutrition.

Focus group discussions with athletes on nutritional adherence and experience of nutrition service delivery were rich and enlightening. For example, while some athletes found body composition assessments a helpful and constructive technique to monitor their dietary behaviour [65], others shared that it can lead to maladaptive dietary behaviour (e.g., restrictive eating) as they felt it reinforced and perpetuated an aesthetically driven culture where their appearance mattered most [33]. Hearing athletes’ individual experience of, and responses to, body composition assessments was influential in developing feelings of compassion and allowed me to shape my own views of such practices. Subsequently, I became attentive to individual differences and preferences towards body composition assessments within Para Sport, as well as how we assessed body composition within the sport.

When I was settling into my *performance* nutrition role in Para Sport, we had developed a “Body Composition Monitoring Policy” where athletes were advised to engage in regular body composition assessments (i.e., every 3 months). Reflecting on the body composition data obtained since I started the role, I noticed there was varying uptake to such guidance across the athlete group (i.e., some would choose to book in regularly to have their DEXA scan others would not at all). Having heard athletes’ views and experiences of body composition monitoring during data collection, I proposed an alternative approach to athletes and the sport science, medicine, and coaching team within Para Sport. When developing this new approach, I asked for their input and opinions on such ideas. Subsequently, we moved away from advising routine body composition monitoring, and instead moved to a process where body composition assessments were decided on a case-by-case basis when clear rationale had been identified by the athlete and multidisciplinary team. I explained the proposed changes to athletes and the multidisciplinary team and asked them, “what am I not seeing?”, “what problem could arise if we were to go ahead in this way?”. Asking for direct feedback facilitated a collaborated approach to revising our process to body composition assessments which was centred on individuality.

Working in a way that serves individuality aligns to the Paralympic movement. Specifically, McNamee and Parnell [60] assert that the paralympic movement is a “celebration of sporting difference” and being clear on differences and distinctions is a central and essential part of paralympic sport’s philological identity. Through engaging in reflective discussions with my supervisory team, it seemed athletes and staff in Para Sport were accustomed to an individualised approach to athlete support, as opposed to a one-size-fits-all strategy. Thus, as a researcher-practitioner I was afforded the opportunity to collaborate with athletes and the multidisciplinary team to devise a process that acknowledged athletes’ individual experiences of body composition assessments as opposed to assuming that athletes share the same perception and therefore should be expected to follow the same protocol, which I had initially done.

High-performance non-disability sporting settings are considered to inhibit the option of athlete choice due to prioritisation of the performance narrative [66], consequently power between athletes and the sport science, medicine, and coaching professionals is a central issue in sport [62,64]. At the beginning of data collection, I felt uneasy and apprehensive engaging in conversations with athletes because of a lack of rapport between us and how athletes would perceive me as a *performance* nutritionist. However, I accepted that the acknowledgement of unequal power between myself and athletes was a necessity to build rapport and breakdown any preconceived ideas of me, and my values and identity. Consequently, being attentive to, and hearing athletes’ individual experiences of a nutritional service, and having the opportunity to adapt my own service in Para Sport gave me confidence that there were diverse ways in which the role can be delivered and received. Reflecting on my journey, it seemed the use of focus group discussions was a vehicle that facilitated a collaborative, inclusive, and person-centred environment as athletes openly voiced their thoughts, feelings, and behaviours. We found working w*ith* athletes to co-design a nutrition service requires communication skills, such as attentive listening, and the belief that there is no one truth to athletes’ experiences. At the time, we did not construct the study design and interview template in partnership with athletes and sport nutritionists, and moving forward we would like to explore this approach as it has been recommended within the co-production literature to address power imbalances [15].

### Practical implications

This unique first-hand account by a sport nutrition researcher-practitioner has several implications for practice. Subsequently, we consider how this process of cultural immersion might inform the development of sport nutrition professional practice within high-performance sport. Firstly, the education of sport nutritionists has primarily focused on nutrition physiology, therefore shifting focus to the learning and development of sport nutrition professionals could help facilitate more holistic understanding of high-performance sport cultures. A shift towards the learning and development of this workforce would acknowledge that learning is a complex, personal and continuous process [67]. We have illuminated the relational and context specific nature of the role, and so any learning and development programme needs to cater for sport nutritionists working in different contexts with different people. Bringing trainees together to explore the nuanced and complex nature of sporting cultures could help inform and influence their delivery of sport nutrition practice.

Secondly, we posit that expressing vulnerability and presenting our unvarnished selves is a key part of learning. However, we recognise the tension that sport nutritionists may experience when expressing vulnerability and seeking credibility. To elaborate, although “making yourself vulnerable” may be good for learning, it exposes us to be less than perfect and can galvanise fear that our peers and colleagues will think less of us. To help practitioners negotiate and embrace this tension we advocate intellectual candour, whereby experienced and neophyte sport nutritionists and their supervisors voice their uncertainties, errors, and concerns to promote a culture of learning that acknowledges fallibility rather than honouring perfectionism [68]. Within this paper we have enacted intellectual candour in a bid to normalise reflexivity and fallibility as a part of sport nutrition professional practice. In turn, we believe this approach will help sport nutritionists to grow and build a complex, holistic view of practice that better serves themselves, their athletes, and colleagues they work with.

Finally, shared reflection was imperative in facilitating the learning and development of the first author throughout her involvement with HPO. The supervisory team helped the first author to understand that sharing and making sense of critical development experiences is invaluable and does not need to be done alone. Therefore, we encourage researchers and/or practitioners to seek out peer support networks and/or mentors/supervisors. Specifically, professional training bodies (e.g., SENR) could support the development of dynamic, interactive, and mutually supportive peer networks across regions. Further, more written accounts of applied practice that advocate the diversity of values and identity in sport nutrition professionals and challenge monolithic thinking within applied practice would extend the sport nutrition field. Engaging in such projects longitudinally would deepen our understanding of how an applied sport nutritionist’s role, values, and identities shape (and are shaped by) applied practice and professional development over time. Such accounts would enrich peer-support network discussions, which in turn would serve to embrace the diverse sport nutrition workforce and ensure that nobody is excluded from developing their practice and enhancing the experience of the people they support.

## Conclusion

This confessional tale provides the first reflexive account of a sport nutrition researcher-practitioner. Within this paper we have illustrated a range of challenges to be(com)ing a sport nutritionist immersed within high-performance sport. Specifically, we have highlighted several practical insights to qualitative data collection gained through cultural immersion and illuminated how reflexivity as a core component of learning and development can strengthen the values and identities of a sport nutrition practitioner. Specially, the reflexive stories shared serve to illuminate the benefits that cultural awareness can bring when developing one’s professional valued and identity. While the sport nutrition field is replete with studies seeking to strengthen our understanding of athletes’ nutritional requirements, there is limited guidance available for *how* practitioners design and deliver their service to increase and sustain the systematic uptake of these guidelines into practice. More research is needed to mend this gap and advance our understanding of reflexivity within sport nutrition professional practice. We demonstrated how the use of a reflexive journal provides an invaluable tool to note and revisit experiences, identify challenges associated with data collection, and highlight research-practitioner vulnerabilities. Embracing vulnerability is not easy, but it serves to flag to ourselves and others that we are open to new ways of knowing.
