# Supplementary material for: A tale of be(com)ing a Sport Nutritionist: Reflexive insights from a researcher-practitioner entering the field
Source: PLoS One. 2025 Feb 27;20(2):e0319164. doi: 10.1371/journal.pone.0319164 (PMC11867323; doi:10.1371/journal.pone.0319164)
Supplement: Attachment — Submitted filename: Response to Reviewers.pdf [file pone.0319164.s002.pdf]

A tale of be(com)ing a Sport Nutritionist: reflexive insights from a researcher-practitioner entering the field

Responses to academic editor and reviewer(s) comments

Comments	Response
Journal Requirements	
1. Please ensure that your manuscript meets PLOS ONE's style requirements, including those for file naming. The PLOS ONE style templates.	We have reviewed the formatting of the manuscript to ensure it meets the style requirement as outlined within the PLOS ONE style templates. Apologies for not following this at the point of submission.
2. We note that you have indicated that there are restrictions to data sharing for this study. PLOS only allows data to be available upon request if there are legal or ethical restrictions on sharing data publicly. For more information on unacceptable data access restrictions, please see	We recognise and support the importance of open and transparent research. For this study, due to the nature of the research and the consenting process, sharing the data is not possible. We have clarified this position within the Data Availability Statement. Please see below:

http://journals.plos.org/plosone/s/data-availability#loc-unacceptable-data-access-restrictions.	Data cannot be shared due to ethical restrictions. Specifically, participants have not agreed to share their data publicly. This is in line with the approval process of Leeds Beckett University Research Ethics Committee.
a) If there are ethical or legal restrictions on sharing a de-identified data set, please explain them in detail (e.g., data contain potentially identifying or sensitive patient information, data are owned by a third-party organization, etc.) and who has imposed them (e.g., a Research Ethics Committee or Institutional Review Board, etc.). Please also provide contact information for a data access committee, ethics committee, or other institutional body to which data requests may be sent.	We have now included the below information within the Data Availability Statement:<br><br>Data cannot be shared due to ethical restrictions. Specifically, participants have not agreed to share their data publicly. This is in line with the approval process of Leeds Beckett University Research Ethics Committee. Contact information for ethical inquiries can be sent to the secretary of the Leeds Beckett University Research Ethics Committee, Dave Clapham at d.p.clapham@leedsbeckett.ac.uk
b) If there are no restrictions, please upload the minimal anonymized data set necessary to replicate your study findings to a stable, public repository and provide us with the relevant URLs, DOIs, or accession numbers. For a list of recommended repositories, please see https://journals.plos.org/plosone/s/recommended-repositories. You also have the option of uploading the	Given the ethical restrictions described within the Data Availability Statement (see above), a description of where the data can be found has not been provided.

data as Supporting Information files, but we would recommend depositing data directly to a data repository if possible.<br><br>We will update your Data Availability statement on your behalf to reflect the information you provide.	
3. Please review your reference list to ensure that it is complete and correct.<br><br>If you have cited papers that have been retracted, please include the rationale for doing so in the manuscript text, or remove these references and replace them with relevant current references. Any changes to the reference list should be mentioned in the rebuttal letter that accompanies your revised manuscript. If you need to cite a retracted article, indicate the article's retracted status in the References list and also include a citation and full reference for the retraction notice.	We apologise for not spotting these errors prior to submission. We have reviewed and amended the reference list to ensure it is complete and compliant with PLOS ONE guidelines for references. Changes to the reference list have been highlighted in blue text. Additionally, no retracted research is included within this manuscript.

Reviewer's Responses to Questions	
1. Is the manuscript technically sound, and do the data support the conclusions?<br><br>The manuscript must describe a technically sound piece of scientific research with data that supports the conclusions. Experiments must have been conducted rigorously, with appropriate controls, replication, and sample sizes. The conclusions must be drawn appropriately based on the data presented.<br><br>Reviewer #1: Yes<br><br>Reviewer #2: Yes<br><br>Reviewer #3: Yes	Thank you for your positive feedback on our work.
2. Has the statistical analysis been performed appropriately and rigorously?<br><br>Reviewer #1: No<br><br>Reviewer #2: N/A<br><br>Reviewer #3: N/A	This research is underpinned by qualitative inquiry, therefore statistical analysis is not applicable, as acknowledged by reviewer 2 and 3.

<table>
<tr><td>3. Is the manuscript presented in an intelligible fashion and written in standard English?

PLOS ONE does not copyedit accepted manuscripts, so the language in submitted articles must be clear, correct, and unambiguous. Any typographical or grammatical errors should be corrected at revision, so please note any specific errors here.

Reviewer #1: Yes

Reviewer #2: Yes

Reviewer #3: Yes</td><td>Thank you for your positive feedback on our work.</td></tr>
<tr><td colspan="2">Review Comments to the Author</td></tr>
<tr><td>Reviewer #1: The English section is not appropriate; you should rephrase or rewrite while all the other things are okay. But the statistical section is not present; there's no proper result and discussion. There's one suggestion: you can add a flow chart for the methodology to improve it.</td><td>This study is underpinned by qualitative inquiry, therefore statistical analysis is not applicable to this research and there are no statistical analyses to present. This manuscript has been written as a results and discussion section combined. Within this format, key findings are critically appraised with pre-existing literature throughout this combined section. This style of write up is well established within the field of qualitative research. We note your suggestion to add a flow</td></tr>
</table>

	chart to visually represent the methodology however, as the research involved an iterative process of data collection and analysis, we would not be able to capture and represent this qualitative approach within a flow chart figure.
Reviewer #2: This is a well-written reflection on entering a new field. The terminology of qualitative research had been well explained. A variety of references had been used, but typing mistakes take away some of the otherwise good narrative.	Thank you for your positive feedback on our work. We apologise for not spotting the typing errors prior to submission. We have reviewed the manuscript to ensure all typing errors are addressed.
Reviewer #3: Regarding statistics, the question seems not to apply once the manuscript was built from a narrative perspective. A personal detail: curiously, I am graduated in both iPhysical Education and Nutrition (with practice and emphasis in sport nutrition). Thus, I believe that the current manuscript is a valuable contribution to the sports nutrition field.	Thank you for your positive feedback and for endorsing the value of our work.
